# Severe non-traumatic bleeding events detected by computed tomography: do anticoagulants and antiplatelet agents have a role?

**DOI:** 10.1186/s13019-014-0166-9

**Published:** 2014-10-15

**Authors:** Olivier Risch, Agaicha Alfidja, Aurelien Mulliez, Anderson H Amani, Louis Boyer, Lionel Camilleri, Kasra Azarnoush

**Affiliations:** pôle Imagerie et Radiologie Interventionnelle CHU de Clermont-Ferrand, ISIT, UMR CNRS 6284 - université d'Auvergne Clermont Hospital, Clermont-Ferrand, France; Bio-statistics unit, Délégation Recherche Clinique & Innovation, Clermont-Ferrand University Hospital, Clermont-Ferrand, France; Heart surgery Department, G. Montpied Hospital, Clermont-Ferrand University Hospital, Clermont-Ferrand, 63000 France; INRA, UMR 1019 Nutrition Humaine, Saint Gen's Champanelle, France

**Keywords:** Intracranial hemorrhage, Muscular hematoma, Anticoagulant, Antiplatelet agents, CT-scan

## Abstract

**Purpose:**

Bleeding is the most common and most serious complication of anticoagulant (AC) and antiplatelet agents (APA) which are increasingly used in every day practice. The aim of this study was to enlist and analyze the most severe bleeding events revealed during computed tomography scanner (CT scan) examinations over a 1-year period at our University Hospital and to evaluate the role of ACs and APAs in their occurrence.

**Methods:**

This descriptive monocentric retrospective study included all patients who benefited from an emergency CT scan with a diagnosis of severe non-traumatic bleeding. Patients were divided into two groups: those treated with ACs and/or APAs, and those not treated with ACs or APAs.

**Results:**

After applying the inclusion criteria, 93 patients were enrolled. Sixty-one patients received an anticoagulant or antiplatelet treatment, and 32 did not receive any AC or APA therapy. Seventy nine percent presented with an intracranial hemorrhage, 17% with a rectus sheath or iliopsoas bleeding or hematoma, and 4% with a quadriceps hematoma. Only patients who received ACs or APAs suffered a muscular hematoma (p <0.0001). Among patients treated with vitamin K antagonists, 6/43 (14%), had an international normalized ratio (INR) higher than the therapeutic range (INR >3).

**Conclusions:**

In our series, intracranial hemorrhage was preponderant and muscular hematomas occurred exclusively in patients treated with ACs and/or APAs. This study needs to be extended to evaluate the impact of new anticoagulant and antiplatelet agents.

**Electronic supplementary material:**

The online version of this article (doi:10.1186/s13019-014-0166-9) contains supplementary material, which is available to authorized users.

## Background

Bleeding is regarded as severe if it has hemodynamic consequences that require transfusion or a hemostatic therapeutic procedure, if vital or functional prognosis is compromised due to its location, or if it cannot be controlled by the usual means. When none of these criteria is present, hemorrhage is qualified as non-severe [1].

Anticoagulants (AC) are the treatment for many pathological conditions, such as venous thromboembolic disease, atrial fibrillation, as well as for patients with mechanical heart valve prosthesis. Although their efficacy has been widely acknowledged, these drugs can lead to complications, the most frequent and severe of which are hemorrhagic events [1].

Currently, more than 900,000 patients receive vitamin-K antagonists (VKA) in France [2]. VKAs are responsible for most iatrogenic complications [3]. In France, the cost of these complications is more than 300 million Euros per year [3] and anticoagulant-related hemorrhagic accidents, especially directly caused by VKAs, are responsible for almost 20.000 hospitalizations per year, with a 5/100 patient-year incidence of severe bleeding events and a 1/100 patient-year incidence of fatal events.

Besides anticoagulants (AC), antiplatelet agents (APA) also induce an important risk for major bleeding. This risk of a hemorrhagic event is nearly doubled when antiplatelet agents are used as a bitherapy [4].

Many factors contribute to the hemorrhagic complications that can occur under anticoagulant therapy [4],[5]. The most important factor is age, with a nearly 50% increased risk of severe hemorrhage per decade in those aged more than 40 years. Arterial hypertension is also a significant risk factor [4]-[6].

Despite the standardization of laboratory procedures, agreement on therapeutic ranges and the introduction of new anticoagulants into the therapeutic arsenal, the occurrence of hemorrhagic events remains significant [7]-[9].

The aim of our study was to register and analyze the most severe bleeding events revealed during CT scan examinations over a 1-year period at our University Hospital and to evaluate the role of ACs and APAs in their occurrence.

## Methods

### Patients

Between April 1, 2011 and March 31, 2012, we performed a monocentric retrospective study that included all patients who had undergone a CT scan for a non-traumatic severe hemorrhage. Exclusion criteria were represented by all suspected traumatic contexts, hemorrhages caused by an underlying tumor, a vascular malformation, or a postoperative bleeding.

During this period, 93 patients were included (50 males, 43 females), mean age 76 ± 11 years, who had been referred by various departments: emergency (n = 75), cardiology (n = 7), recovery unit (n = 5), rheumatology (n = 2), infectious-disease department (n = 2), and internal medicine (n = 2).

Seventy-three patients underwent a cerebral, 16 an abdominal, and 4 a lower-limb CT scan. Data were collected using Xplore Exploitation software, version 6.2.814 (EDL, Santé La Seine sur Mer, France), which records all reports from CT scans performed in our Center (22,076 over this period), and this information was then complited with the patients' hospital-examination files. The studied parameters are listed in Table 1.

**Table 1:** **Indications and results of CT-scans in patients with a hemorrhagic event**

Indications and results of CT-scans	Number	Percentage
Consciousness disorders	39	42
Headache	13	14
Focal neurological signs	59	63
Abdominal pain	15	16
Anemia	21	24
Thigh swelling	4	4
**Results**		
**Cerebral CT-scan (intracranial hemorrhage)**	73	78
Subarachnoid hemorrhage	5	
Peri-cerebral hematoma	12	
Intraparenchymal hematoma	62	
Intraventricular hemorrhage	34	
Cerebral herniation	36	
**Abdominal and lower-limb CT-scan (muscular hematoma)**	20	22
Rectus abdominis hematoma	11	
Iliopsoas hematoma	5	
Quadriceps hematoma	4	

The 93 patients were divided into two groups: group 1, those who did not received ACs or APAs (n = 32) and group 2, those who were treated with ACs and/or APAs (n = 61) (Table 2). Details on the AC and APA treatments are presented in Table 3. Thirty eight patients received ACs, 16 received APAs, 6 received both ACs and APAs, and one patient received two APAs. The AC and/or APA indications were atrial fibrillation (39%), thromboembolic disease (13%), coronary-artery disease (12%), mechanical prosthesis (5%), and a previous history of stroke (5%).

**Table 2:** **Comparison between patients treated with anticoagulants (AC) or antiplatelet agents (APA) and those not treated with either**

	Group 1:	Group 2:	***p-value***
	Not treated:***n*** = 32	AC or APA:***n*** = 61	
Intracranial hemorrhage	32 (100)	41 (67)	NA
Cerebral herniation (mm)	7.1 ± 2.7	9. ± 4.6	0.04
Muscular hematoma	0 (0)	20 (33)	<0.0001
Rectus abdominis, iliopsoas hematoma	0 (0)	16 (24)	0.001
Quadriceps hematoma	0 (0)	4 (7)	0.30
Muscular hematoma (mL)	NA	363 ± 349	NA
Anemia (Hb <10 g/dL)	3 (3)	18 (30)	0.02
Hematocrit (%)	39.3 ± 6	35.6 ± 8.2	0.04
Blood platelets	221 ± 64	247 ± 88	0.28
Transfusion of globular sediment	1 (3)	7 (11)	0.17
Death	5 (15)	17 (28)	0.19
Delay between CT-scan and death (days)	13.4 ± 21.1	7.8 ± 19.9	0.04
In-hospital stay period of surviving patients (days)	27.7 ± 34.3	19.8 ± 26.4	0.35

Data are mean ± standard deviation, median (interquartile range) or number (%).

*Abbreviations*: *mm* millimeter, *mL* milliliter, *g*/*dL* gram per deciliter, *NA* not applicable.

**Table 3:** **Prescribed anticoagulant and antiplatelet agents**

Anticoagulant treatments and antiplatelet agents	Number	Percentage
**Anticoagulants**	37	40
Acenocoumarol	8	
Fluindione	21	
Coumadin	4	
Unfractionated heparin	1	
Low molecular weight heparin	3	
**Antiplatelet agents**	17	18
Aspirin	13	
Ticlopidine	1	
Clopidogrel	3	
**Antiplatelet bitherapy**	1	1
Aspirin, clopidogrel	1	
**Anti-vitamin-K + antiplatelet agents**	6	6
Fluindione + aspirin	5	
Coumadin + aspirin	1	

Anticoagulation reference thresholds for the international normalized ratio (INR) were between 2.5 and 3.5 for mitral mechanical prosthesis and between 2 and 3 for the other indications.

This descriptive, retrospective monocentric study was conducted according to ethical principles for medical research involving human subjects in French university hospitals [10].

### Statistical analyses

The statistics were computed using STATA V10 software (Stata Corp, College Station, Texas, USA). Data are expressed as frequencies and associated percentages for categorical data, and as means ± standard deviations for continuous data.

The categorical data from the two treatment groups were compared using the chi-squared test (or Fisher's exact test, if necessary). Continuous data were compared using Student's *t*-test (or the Kruskal-Wallis test, if necessary), normality was verified by the Shapiro-Wilk test and homoscedasticity by the Fisher'Snedecor test.

The risk ratio, for patients who received AC or APA, for a cerebral hemorrhage and subsequent cerebral herniation, a rectus sheath, iliopsoas bleeding, or a quadriceps hematoma were shown with their corresponding 95% confidence intervals.

All tests were two-sided, with the type 1 error set at α = 0.05.

## Results

According to international guidelines and our university hospital's heart-surgery department's recommendations [4],[11],[12] the indications for AC and APA treatments were all justified. APA treatments were prescribed mainly for coronary and/or peripheral arteriosclerosis; ACs, unfractionated heparin, Low Molecular Weight Heparin (LMWH) and VKAs were prescribed for thromboembolic disease, atrial fibrillation or in postoperative orthopedic patients.

Four patients had an INR between 3 and 4, one patient between 4 and 6, and another had a ratio >6. Among these patients, 2 patients had a muscular hematoma and 4 patients had an intracranial hemorrhage. Comparisons between patients treated and not treated by AC and APA are listed in Table 2.

Muscular hematoma occurred in 22% of cases (n = 20), among which 17% (n = 16) were of the rectus sheath or iliopsoas, and 4% (n = 4) were quadriceps hematomas. Muscular bleedings and hematomas occurred exclusively in patients treated by AC or APA. This difference was statically significant (p <0.0001) (Table 2).

**Figure 1:**
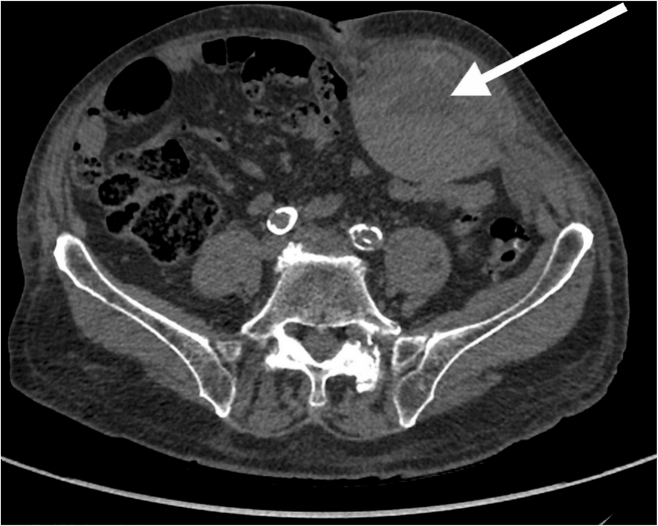
**Abdominal CT scan.** Hematoma of the rectus abdominis (White arrow).

**Figure 2:**
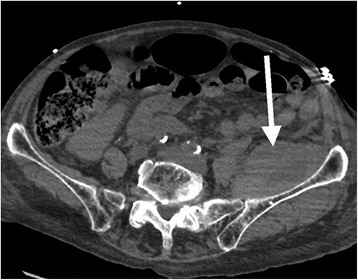
**Abdominal CT scan.** Hematoma of the left iliopsoas (White arrow).

All patients (n = 4) with a quadriceps hematoma received an anticoagulation treatment (AC exclusively in 3 patients, and AC + APA in 1 case) (Table 4). Five cases of iliopsoas hematomas were recorded (all in patients treated by AC), and 11 patients had a rectus sheath hematoma (9 received AC, 1 had APA, and 1 had a bitherapy of AC + APA) (Table 4). The estimated risk of soft tissue hematoma was 3.9 [1]-[15] in patients who received AC compared to patients treated by antiplatelet therapy (Figures 1 and 2).

**Table 4:** **Incidence of hemorrhages with the different treatments: anticoagulants (AC) and/or antiplatelet agents (APA), or no drug therapy**

	Not treated:***n*** = 32	Only AC:***n = 38***	APA:***n*** = 16	AC + APA:***n*** = 6	Double APA:***n*** = 1
Intracranial hemorrhage	32 (100)	21 (55)	15 (94)	4 (67)	1 (100)
Muscular hematoma: *n* = 20	0 (0)	17 (45)	1 (6)	2 (33)	0 (0)
Quadriceps (*n* = 4)	0 (0)	3 (8)	0 (0)	1 (17)	0 (0)
Rectus abdominis (*n* = 11)	0 (0)	9 (24)	1 (6)	1 (17)	
Iliopsoas (*n* = 5)	0 (0)	5 (13)	0 (0)	0 (0)	0 (0)
Cerebral-hematoma volume (mL)	42.9 ± 45.5	65.8 ± 46.4	28 ± 37.7	23.3 ± 44.5	0(0)
Transfusion: *n* = 8	1 (3)	7 (18)	0 (0)	0 (0)	0 (0)
Anemia (Hb <10 g/dL): *n* = 21	3 (9)	15 (39)	1 (6)	2 (33)	0 (0)
Hematocrit (%)	39.3 ± 6	33.8 ± 9.2	39.4 ± 4.2	34.9 ± 7.9	MD
Deaths: *n* = 22	5 (16)	13 (34)	4 (25)	0 (0)	0 (0)
Delay between CT-scan and death (days)	13.4 ± 21.1	2.8 ± 5	24 ± 39.5	0 (0)	0 (0)
In-hospital stay (days)	15 [3 – 37]	11.5 [6 – 19.5]	12 [6 – 24]	15 [4 – 78]	2 [2 – 2]

Data are mean ± standard deviation, median (interquartile range) or number (%). MD: Missing Data.

*Abbreviations*: *mm* millimeter, *mL* milliliter, *g*/*dL* gram per deciliter, *NA* not applicable.

The average muscular hematoma volume was 363 ± 249 mL, and was associated with a statistically significant decrease in hemoglobin and hematocrit (p 0.02 and 0.04, respectively) (Table 2).

An intracranial hemorrhage (n = 73) was the most common bleeding event, 78% of the observed bleedings (0.03% of the total 2274 emergency cerebral CT scans performed during this period). A cerebral hemorrhage was observed in 67% (n = 41) of patients treated with AC and/or APA (Table 2).

Among patients with an intracranial hemorrhage, 32 (44%) received neither AC nor APA treatment, 21 had a single AC treatment, 15 had a single antiplatelet therapy, 4 had a bitherapy (AC + APA), and 1 had a double antiplatelet therapy (Table 4).

**Figure 3:**
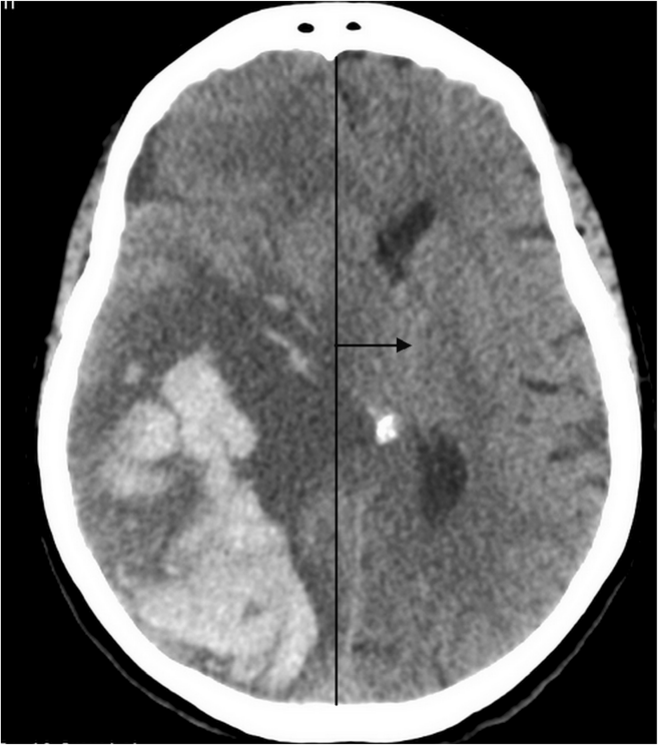
**Cranial CT scan.** Right parietal occipital hematoma with cerebral oedema and left displacement of the medial brain structures (Black arrow).

Cerebral herniation, estimated by the displacement of medial brain structures (Figure 3), was significantly more important in patients treated by AC and/or APA (9.8 ± 4.6 mm) compared to group 1 patients (7.1 ± 2.7 mm; p = 0.04). The risk of cerebral herniation was higher in patients who received AC compared to patients who received a single APA therapy (62% vs. 31%, respectively, RR = 2.3 [0.9–5.7]).

Among the 93 patients, 22 died (24%). In 19 cases (86%) the cause of death was an intracranial hemorrhage and of these, 14 (74%) were treated with AC or APA.

The time until death was statistically shorter for group 2 patients compared with group 1: 7.8 ± 19.9 vs. 13.4 ± 21,1 days, p = 0.04 (Table 2) and it was even shorter for patients who received AC (2.8 ± 5 days) when compared to all other treatments. Patients who had a bitherapy (AC + APA or APA + APA) were all alive at the end of this study (Table 4).

## Discussion

In our series, which included patients who had suffered a severe hemorrhage detected by CT scan, the intracranial location was predominant. In addition, these intracranial hemorrhages were more severe when patients received AC or APA therapy. Muscular bleedings and hematomas were not observed in patients who did not receive any AC or APA.

In our patient population, the main fatal hemorrhagic event was intracranial hemorrhage (86%). In France these account for 10–15% of the total number of cerebrovascular events, 10–20 cases per 100,000 inhabitants [6]. Their incidence per year is between 0.3 and 0.6%.

Palareti et al., [13] in a series that included severe as well as minor hemorrhages, reported 153 hemorrhagic complications (7.6 per 100 patient-years), of which five were fatal (all cerebral hemorrhages, 0.25 per 100 patient-years), 23 were major (1.1), and 125 were minor (6.2).

Our study did not find any significant difference in the occurrence of intracranial hemorrhage depending on whether the patient received ACs or not; however, worsening elements of a cerebral hemorrhage, such as the volume of the cerebral hematoma, were statistically significant in patients receiving ACs.

Cucchiara et al.'s study [14] confirms this finding. In their series, the initial volume, the progression, and the mortality rate from intracranial hemorrhage were greater in patients who received VKAs compared to those who did not receive ACs.

Tonolini et al. [15] reported that bleeding occurred in almost every muscle group in patients treated with ACs or APAs. On CT scan, the appearance of muscular hemorrhages depends on its location, and bleeding rate. Rectus sheath and iliopsoas are the most common locations. Patients usually complain with acute abdominal pain and distension, associated with a fall in hematocrit and often a hypovolemic shock. In our series, severe muscular hematomas that required a CT scan occurred exclusively in patients who had received AC or APA therapy.

Venous thromboembolic disease and prevention of cardiac arterial emboli during atrial fibrillation are the two main indications for prescribing ACs [1],[4],[16]. In our study, atrial fibrillation and thromboembolic venous disease were the most frequent indications, accounting for, respectively, 38.5% and 13% of the cases. In a study that included 188,740 elderly patients (aged >65 years), Van Walraven et al. [17] reported that 10,020 people (5.3%) were treated with VKAs (3.5% of the total population). People who received ACs had a mean age of 77 years, and 50% were men. They spent 26.7% of their VKA exposure time with an INR <2 and 14.2% of the time with an INR > 3. They had an overall hemorrhagic rate of 6% per year. Bleeding rates increased significantly when INR exceeded 3. As reported by Butchart [17] important INR variations are as important as the high level of INRs to explain some hemorrhagic accidents due to vitamin k antagonists and only a careful INR control with dedicated devices can decrease bleeding complications due to this variations [18].

Old age is a risk factor for hemorrhagic bleedings in patients receiving ACs or APAs [1],[4],[16],[19], particularly in those aged >75 years, but also for patients with a prior history of intracranial hemorrhage [19].

Many studies show that there is a high association between age and hemorrhagic risk. In our series, cerebral hemorrhagic events occurred in 62.3% of patients aged >65 years, but there was no statistically significant difference in younger patients. Similarly, the role of arterial hypertension, as well as described by some authors [5],[6], was not evidenced in our experience. This could be related to our study population, which included only severe hemorrhagic events detected by CT scan. After AC treatment, arterial hypertension is the second most important risk factor for intracranial hemorrhage occurrence [20],[21]. Alcohol and tobacco are other risk factors [20].

### Study limitations

This descriptive study is a collection of severe hemorrhagic events, detected by a CT-scan in our Center, without a comparative control population. In addition, our selection method, which only included those who benefited from a computerized CT scan, could have excluded patients with milder hemorrhagic events which did not require a CT scan, and also rapidly mortal hemorrhages. The timeframe of our monocentric study was too short to allow exhaustive analysis. A multicenter study with a longer follow-up period is needed to confirm our preliminary results.

## Conclusion

Severe hemorrhages are preponderantly intracranial and have a high mortality rate, regardless of the patient's exposure to anticoagulant or antiplatelet agents. This study confirms that muscular and soft-tissue hemorrhagic events exclusively occur in patients who received AC and/or APA therapies. Further investigations are needed to monitor the effects of new anticoagulant and antiplatelet agents.

## Authors' contributions

OR did the data acquisition and participated in manuscript conception. AA did the data interpretation and participated in manuscript design. AM performed the statistical analysis. AHA did data acquisition. LB and LC participated in study conception and did the final approval. KA: conceived the study, and participated in its design and coordination and helped to draft the manuscript. All authors read and approved the final manuscript.

## Authors’ original submitted files for images

Below are the links to the authors’ original submitted files for images. Authors’ original file for figure 1 Authors’ original file for figure 2 Authors’ original file for figure 3

## Abbreviations

AC: Anticoagulants
APA: Antiplatelets agents
CT scan: Computed Tomography scanner
INR: International Normalized Ratio
VKA: Vitamin K antagonists
LMWH: Low molecular weight heparin

## References

[1] Dunning J, Versteegh M, Fabbri A, Pavie A, Kolh P, Lockowandt U, Nashef SA (2008). Guideline on antiplatelet and anticoagulation management in cardiac surgery. Eur J Cardiothorac Surg.

[2] Gras-Champel V, Brenet-Dufour V, Moragny J, Masson H, Davidau E, Masmoudi K, Andrejak M (2010). Quantification of the part allocated to the preventability of vitamin K antagonists therapy bleeding events. Therapie.

[3] Pouyanne P, Haramburu F, Imbs JL, Bégaud B (2000). Admissions to hospital caused by adverse drug reactions: cross sectional incidence study. French Pharmacovigilance Centres. BMJ.

[4] Lansberg MG, O'Donnell MJ, Khatri P, Lang ES, Nguyen-Huynh MN, Schwartz NE, Sonnenberg FA, Schulman S, Vandvik PO, Spencer FA, Alonso-Coello P, Guyatt GH, Akl EA (2012). American College of Chest Physicians. Antithrombotic and thrombolytic therapy for ischemic stroke: Antithrombotic Therapy and Prevention of Thrombosis, 9th ed: American College of Chest Physicians Evidence-Based Clinical Practice Guidelines. Chest.

[5] Cosserat F, Colnat-Coulbois S, Klein O, Audibert G, Petitpain N, Tréchot P, Auque J (2009). Hémorragies intracrâniennes et anticoagulants oraux: étude des facteurs pronostiques à partir d'une série de 186 cas. Neurochirurgie.

[6] Grillo P, Velly L, Bruder N (2006). Accident vasculaire cérébral hémorragique: nouveautés sur la prise en charge. Ann Fr Anesth Reanim.

[7] Poli D, Antonucci E, Testa S, Tosetto A, Ageno W, Palareti G (2011). Result of a prospective collaborative study on elderly patients followed by Italian centres for anticoagulation. Circulation.

[8] Connolly SJ, Ezekowitz MD, Yusuf S, Eikelboom J, Oldgren J, Parekh A, Pogue J, Reilly PA, Themeles E, Varrone J, Wang S, Alings M, Xavier D, Zhu J, Diaz R, Lewis BS, Darius H, Diener HC, Joyner CD, Wallentin L (2009). Dabigatran versus Warfarin in Patients with Atrial fibrillation. N Engl J Med.

[9] Patel MR, Mahaffey KW, Garg J, Pan G, Singer DE, Hacke W, Breithardt G, Halperin JL, Hankey GJ, Piccini JP, Becker RC, Nessel CC, Paolini JF, Berkowitz SD, Fox KA, Califf RM (2011). Rivaroxaban versus warfarin in nonvalvular atrial fibrillation. N Engl J Med.

[10] Claudot F, Alla F, Fresson J, Calvez T, Coudane H, Bonaïti-Pellié C (2009). Ethics and observational studies in medical research: various rules in a common framework. Int J Epidemiol.

[11] Azarnoush K, Camilleri L, Aublet-Cuvelier B, Geoffroy E, Dauphin C, Dubray C, De Riberolles C (2011). Results of the first randomized French study evaluating self-testing of the International Normalized Ratio. J Heart Valve Dis.

[12] Coppens M, Eikelboom JW, Hart RG, Yusuf S, Lip GY, Dorian P, Shestakovska O, Connolly SJ (2013). The CHA2DS2-VASc score identifies those patients with atrial fibrillation and a CHADS2 score of 1 who are unlikely to benefit from oral anticoagulant therapy. Eur Heart J.

[13] Palareti G, Leali N, Coccheri S, Poggi M, Manotti C, D'Angelo A, Pengo V, Erba N, Moia M, Ciavarella N, Devoto G, Berrettini M, Musolesi S (1996). Bleeding complications of oral anticoagulant treatment: an inception-cohort, prospective collaborative study (ISCOAT). Italian Study on Complications of Oral Anticoagulant Therapy. Lancet.

[14] Cucchiara B, Messe S, Sansing L, Kasner S, Lyden P (2008). Hematoma Growth in Oral Anticoagulant Related Intracerebral Hemorrhage. Stroke.

[15] Tonolini M, Ippolito S, Patella F, Petullà M, Bianco R (2012). Hemorrhagic complications of anticoagulant therapy: role of multidetector computed tomography and spectrum of imaging findings from head to toe. Curr Probl Diagn Radiol.

[16] Bauersachs RM (2012). Use of anticoagulants in elderly patients. Thromb Res.

[17] Butchart EG, Payne N, Li HH, Buchan K, Mandana K, Grunkemeier GL (2002). Better anticoagulation control improves survival after valve replacement. J Thorac Cardiovasc Surg.

[18] Azarnoush K, Dorigo E, Pereira B, Dauphin C, Geoffroy E, Dauphin N, D'Ostrevy N, Legault B, Camilleri L (2013). Mid-term results of self-testing of the international normalized ratio in adults with a mechanical heart valve. Thromb Res.

[19] Van Walraven C, Oake N, Wells PS, Forster AJ (2007). Burden of potentially avoidable anticoagulant-associated hemorrhagic and thromboembolic events in the elderly. Chest.

[20] Feldmann E, Broderick JP, Kernan WN, Viscoli CM, Brass LM, Brott T, Morgenstern LB, Wilterdink JL, Horwitz RI (2005). Major risk factors for intracerebral hemorrhage in the young are modifiable. Stroke.

[21] Brott T, Thalinger K, Hertzberg V (1986). Hypertension as a risk factor for spontaneous intracerebral hemorrhage. Stroke.

