# Supplementary figures and images for: Severe non-traumatic bleeding events detected by computed tomography: do anticoagulants and antiplatelet agents have a role?
Source: J Cardiothorac Surg. 2014 Oct 15;9:166. doi: 10.1186/s13019-014-0166-9 (PMC4200130; doi:10.1186/s13019-014-0166-9)

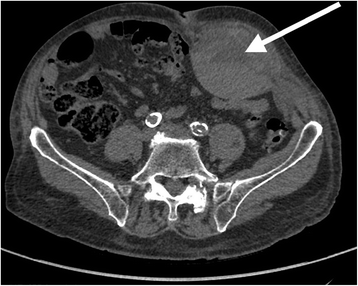

Supplement: Supplementary file 1 — Authors’ original file for figure 1 [file 13019_2014_166_MOESM1_ESM.gif]

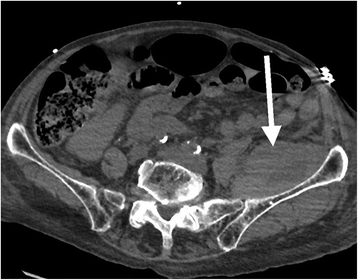

Supplement: Supplementary file 2 — Authors’ original file for figure 2 [file 13019_2014_166_MOESM2_ESM.gif]

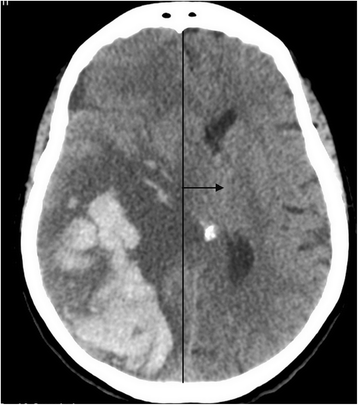

Supplement: Supplementary file 3 — Authors’ original file for figure 3 [file 13019_2014_166_MOESM3_ESM.gif]
